# Rheumatoid Arthritis Disadvantages Younger Patients for Cardiovascular Diseases: A Meta-Analysis

**DOI:** 10.1371/journal.pone.0157360

**Published:** 2016-06-16

**Authors:** Jaap Fransen, Seyyed M. R. Kazemi-Bajestani, Sebastian J. H. Bredie, Calin D. Popa

**Affiliations:** 1 Department of Rheumatology Radboud University Nijmegen Medical Centre, Nijmegen, The Netherlands; 2 Department of Oncology, Faculty of Medicine, University of Alberta, Edmonton, Canada; 3 Department of Internal Medicine, Radboud University Nijmegen Medical Centre, Nijmegen, The Netherlands; 4 Department of Rheumatology, Bernhoven Hospital, Uden, The Netherlands; Boston University, UNITED STATES

## Abstract

**Introduction:**

The incidence of cardiovascular diseases (CVD) is increased in rheumatoid arthritis (RA) patients. It remains unclear whether the load of RA increases cardiovascular (CV) risk especially in female and in younger RA patients. In the present study we aim to analyse the influence of age and gender on CV risk in RA relative to the general population, using meta-analysis of direct comparative studies.

**Method:**

Systematic literature search was performed in MEDLINE for studies reporting on occurrence of CV events in RA as compared to the general population, stratified for gender and/or age. Quality was appraised using the Newcastle-Ottawa scale. Meta-analysis was performed on rate ratios using inverse variance methods.

**Results:**

There were 1372 records screened and 13 studies included. RA females and males have a similar higher risk (95%CI) to develop stroke with RR 1.35 (1.30–1.40) and RR 1.31 (1.21–1.43); coronary artery disease with RR 1.65 (1.54–1.76) versus RR 1.55 ((1.41–1.69) in men; cardiovascular disease with RR 1.56 (1.49–1.62) versus 1.50 (1.41–1.60). The highest incidence of CV events was observed in the youngest patients, RR 2.59 (1.77–3.79), whereas older patients had the lowest relative risk when compared to the general population, RR 1.27 (1.16–1.38).

**Conclusion:**

The relative risk of RA patients for CVD is age dependent, but does not depend on gender: the relative risk on CVD appears to be equally raised for males and females, while relatively young RA patients (<50 years) have the highest, and older patients the lowest relative risk.

## Introduction

It is well-known that CV risk in RA is increased, and that the ‘excess’ risk of CVD in RA is not explained by ‘traditional’ risk factors [1–4]. RA itself may lead to an increased risk of CVD, presumably through systemic inflammation. In the general population, CV risk is higher in males compared to females, and CV risk increases with age [5]. However, no study has confirmed this dogma in RA population yet. It may therefore be that the load of RA modulates the impact of age and gender on CV risk in RA following other determinants as compared to the general population. Indeed, some data suggest that men and women with RA are not equally affected by inflammation with respect to CV risk factors. In line with this, we have previously shown that HDL-2 subfraction is mostly declined in RA women, whereas no differences have been observed in men with RA [6]. It has been demonstrated that early menopause is a risk factor for developing RA [7] and a recent study showed that RA patients with a history of early menopause before disease onset have an increased risk of CVD [8]. Moreover, due to active inflammation, RA women are predisposed to reach menopause earlier in life, augmenting their CV risk [9,10]. Finally, low-grade inflammation and a disturbed metabolism, as it is the case in diabetes mellitus, may augment the CV risk much more in women (relative risk 3 to 8 times higher) than in men (relative risk 2 to 3 times higher) as compared to the general population [11]. Therefore, the question arises whether and in how far, such differences would translate into a different distribution of the relative risk of CVD in RA based on gender and age, compared to the general population. Knowledge about this distribution of the excess CVD risk over age and gender may eventually contribute to improved risk prediction in RA.

The objective of the present study is to analyse the influence of age and gender on CV relative risk in RA patients as compared to the general population, using meta-analysis of direct comparative studies.

## Patients and Methods

This is a systematic literature search with meta-analysis of cohort studies. A systematic literature search of the MEDLINE (via PubMed) database (1980 to December 2013) was performed to identify studies reporting on the relative risk of cardiovascular events in RA patients, as compared to people from the general population, stratified by gender and/or age. Results were pooled in meta-analysis, stratified by age and gender and CV outcome.

### Search

The search strategy was performed together with a librarian, using Rheumatoid Arthritis (MESH or title/abstract), combined with: stroke, myocardial infarction, cardiovascular diseases, cerebrovascular accident, (MESH or title/abstract) combined with: men, women or male, female (MESH and title/abstract), and a limitation to English language. Reference lists of systematic reviews were screened for potential eligible studies.

### Study selection

One author (C.P.) screened all titles and abstracts of the search result for potential inclusion. Two authors (C.P. and S.B.) then independently read the full text of the selected studies for inclusion, according to the in- and exclusion criteria. Disagreements were resolved by consensus. If consensus was not reached, another author (J.F.) was asked to read the study and a final decision was made by consensus.

Studies were included if: the relative risk of CV events was assessed in a cohort of RA patients in comparison with a control cohort from the general population; if the CV outcome included myocardial infarction and/or stroke, or mortality due to CV events; if CV risk was separately reported for males and females and/or for different age groups. Studies were excluded if no comparison between RA patients and controls was made or could be made from the data available, if data were not stratified by age and/or gender, if less than 100 patients were included; if it were reviews or meta-analyses; if written in a language other than English.

### Data extraction and quality assessment

One investigator (J.F.) systematically extracted the following data from each study for all age groups and gender by type of outcome: standardized mortality ratio, standardized morbidity ratio, or incidence rate ratio, their components, group size, 95% confidence interval, standard error, or p-value. All included studies were analyzed in meta-analysis for the CV outcomes presented. However, not all studies contributed data for the same CV outcomes (e.g. if data were present on coronary artery disease but not for stroke) and some studies had data on multiple outcomes (e.g. on coronary artery disease as well as on stroke) or combined outcomes (e.g. coronary artery disease and/or stroke). In S1 Table it can be seen for which study which data were present.

To indicate the risk of bias, all selected studies were assessed for quality at study level by two reviewers (J.F. and C.P.) independently using the Newcastle-Ottawa Scale; cohort studies can receive a maximum of 4 points (‘stars’) for Selection, 2 points for Comparability, and 3 points for Outcome [12]. Disagreement in quality assessment was solved by discussion.

### Data synthesis and statistical analysis

Extracted data were combined for meta-analysis using Review Manager (RevMan 5.3) software (Cochrane Collaboration). Available estimates of relative risk were combined using the inverse variance method in a random effects model [13]. Forest plots were constructed to summarize the relative risk estimates and their 95% CIs, comparing the risk of RA patients with the risk in people from the general population, by age groups and gender, for each type of cardiovascular outcome. Heterogeneity across studies was assessed using I^2^.

## Results

### Study selection

**Fig 1:**
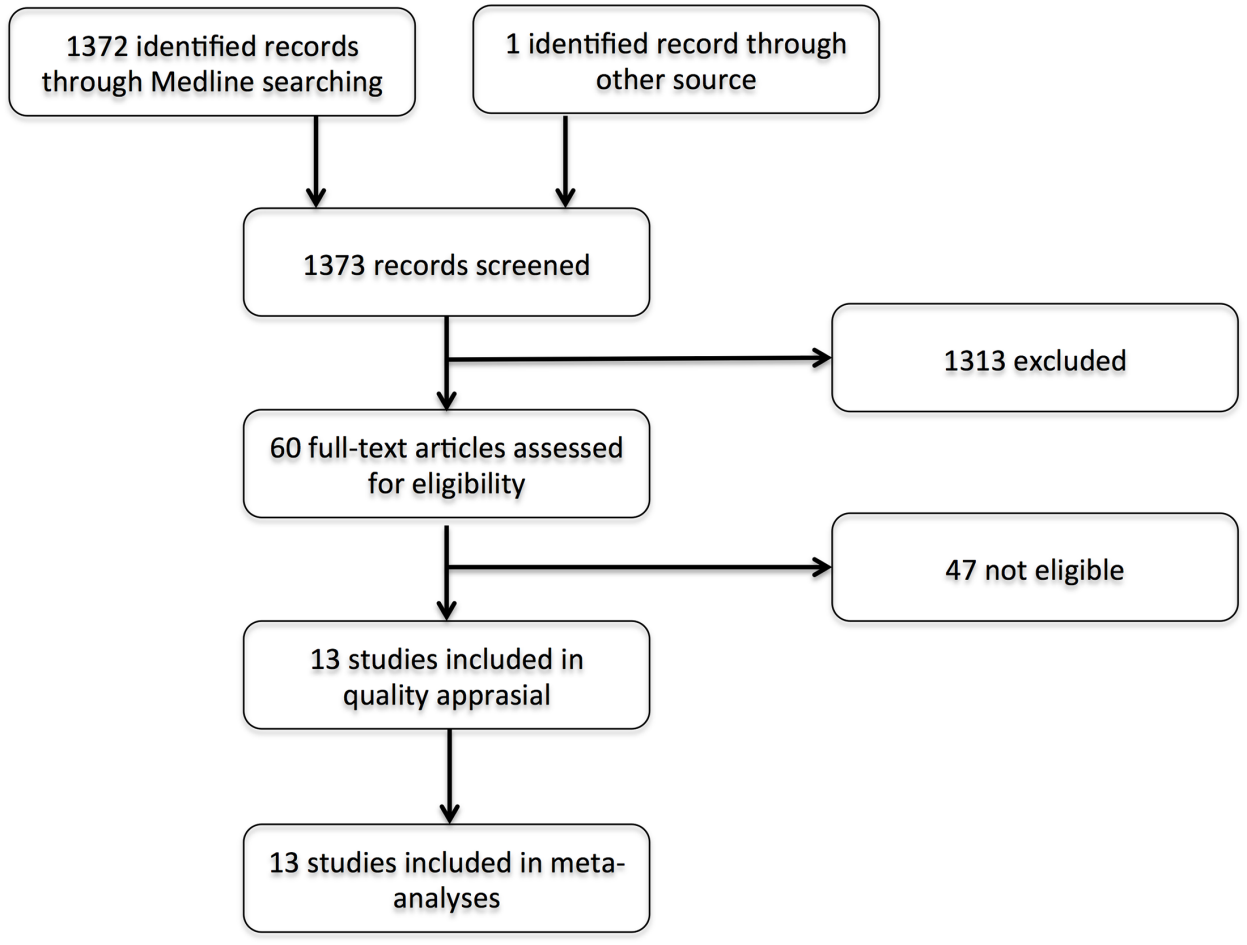
Flow diagram of the published articles evaluated for inclusion in this meta-analysis.

We identified 1372 unique references through searching the literature databases (Fig 1). Based on screening of title and/or abstract, 60 references were selected for full-text review. There were 47 studies excluded for the following reasons: cross-sectional study (5); case-control study (1); no control group from general population (13); no appropriate data on age/sex comparison (26); no data on cardiovascular disease (1); no full-text available (1). Consequently, 13 studies were eligible for meta-analysis (Table 1): eight including stroke, seven including myocardial infarction (CAD), eleven including a combination of stroke and CAD, or CVD in general, and four included cardiovascular death as outcomes [14–26].

**Table 1:** Sample size, female percentages and mean age of RA patients participating in the studies included in this meta-analysis.

Study (cohort)	Sample size (n)		Female	Age
	RA	Non-RA		
^14^ Semb et al. *(AMORIS study*, *Sweden)*	1 779	478 627	71%	57.7(14.0)^a^
^15^ Turreson et al. *(Malmo inhabitants*, *Sweden)*	1 022	207 846	74%	n.a.
^16^ Lindhardsen et al. *(Danish nationwide cohort)*	18 247	4 164 088	70%	59.2(14.1)
^17^ Lindhardsen et al. *(Danish nationwide cohort)*	9 921	3 978 821	71%	56.4(15.5)
^18^ Solomon et al. *(British Columbia inhabitants)*	25 385	252 976	71%	n.a.
^19^ Norton et al. *(Early RA Study—ERAS; UK)*	1 460	n.a.	66%	55.3(14.6)
^20^ Holmqvist et al. *(National Patient Register and Swedish Population Register; Sweden)*	39 065	171 965	73%	n.a.
^21^ Bergström et al. *(1978 cohort*, *Malmo Sweden)*^b^	148	n.a.	79%	46.3(14.2)
^21^ Bergström et al. *(1995 cohort*, *Malmo Sweden)*	161	n.a.	78%	48.3(16.4)
^22^ Goodson et al. *(The Norfolk Arthritis Register—NOAR; UK)*	575	n.a.	68%	57(45–68)
^23^ Wållberg-Jonsson et al. *(Vasterbotten*, *Sweden)*	606	n.a.	68%	56(17–83)^a^
^24^ Myllykangas-Luosujärvi et al. *(Finnish nationwide)*	1666	n.a.	71%	n.a.
^25^ Watson et al. *(General Practice Research Database—GPRD; UK)*	11 633	2 373 551	70%	n.a.
^26^ Thomas et al. *(Scottish inpatient records)*	41 344	n.a.	69%	n.a.

Studies are described by reference number and first author name; Age: mean (SD) in years; n.a. = not available; the studies without control non-RA groups used age and gender-specific mortality rates obtained from their national statistics offices (reference 19, 21, 22, 23, 24, 26).

^a^age for female group;

^b^in the study of Bergström et al. there were two cohorts investigated: from 1978 and 1995.

### Quality appraisal

Of the 13 included studies (Table 2), 9 received maximal scores for all three domains: selection, comparability, and outcome; while 1 study lost 1 point in the selection domain because it was not reported that the outcome (CVD) always occurred after onset of RA, 2 of confounding factors other than age and gender, and 4 studies lost one point in the outcome domain, mostly because of no statement on subjects being lost to follow-up.

**Table 2:** Critical appraisal using the Newcastle-Ottawa Quality Assessment Scale.

[Ref]	Study	Selection	Comparability	Outcome
24	Myllykangas, 1995	✰✰✰✰	✰	✰✰
23	Wallberg, 1997	✰✰✰	✰	✰✰
22	Goodson, 2002	✰✰✰✰	✰✰	✰✰✰
26	Thomas, 2003	✰✰✰✰	✰✰	✰✰✰
25	Watson, 2003	✰✰✰✰	✰✰	✰✰✰
15	Turesson, 2004	✰✰✰✰	✰✰	✰✰✰
18	Solomon, 2006	✰✰✰✰	✰✰	✰✰✰
21	Bergström, 2009	✰✰✰✰	✰✰	✰✰
14	Semb, 2010	✰✰✰✰	✰✰	✰✰
17	Lindhardsen, 2011	✰✰✰✰	✰✰	✰✰✰
20	Holmqvist 2012	✰✰✰✰	✰✰	✰✰✰
16	Lindhardsen, 2012	✰✰✰✰	✰✰	✰✰✰
19	Norton, 2013	✰✰✰✰	✰✰	✰✰✰

The Newcastle-Ottawa Quality Assessment Scale for cohort studies awards a maximum of four points for ‘selection’, two points for ‘comparability’ and three points for ‘exposure’ [10].

### Influence of gender on CV events

**Fig 2:**
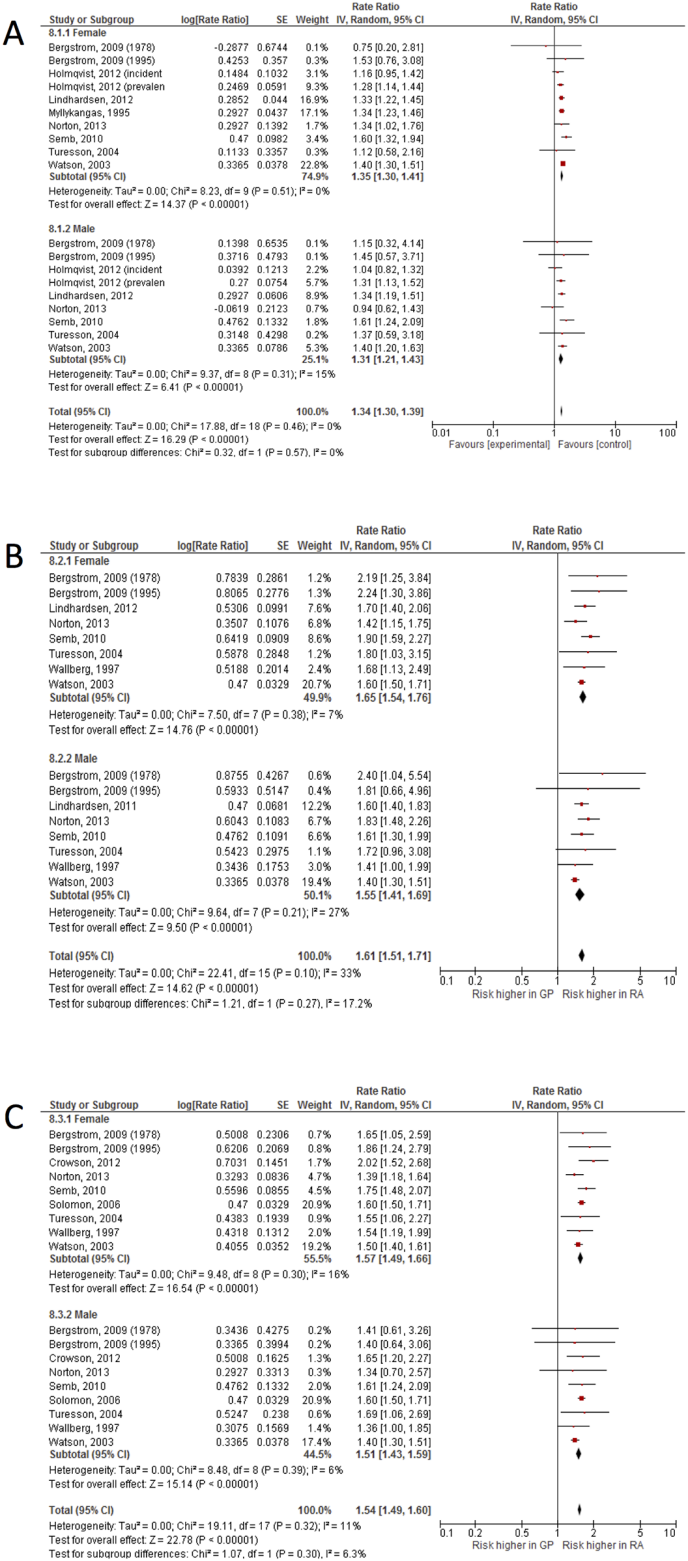
The effect of gender on different CV events. Similar risk of stroke (A), coronary artery disease (B) and CVD in general (C) among women and men with RA.

In 12 of the 13 included studies, CVD risk of RA patients was compared to people from the general population for males and females separately, one study reported only about females (see S1 Table for details). Nearly all studies showed a higher relative risk on stroke and CAD for both male and female patients with RA (Fig 2). Using these studies, the overall relative risk for patients with RA, in comparison to people from the general population, was 1.34 for stroke, 1.61 for CAD, and 1.53 for combined CVD events (Fig 2A–2C) and 1.57 for CVD mortality. Both RA females and males had a similar relative risk (p = 0.57) to develop stroke when compared to the general population: RR 1.35 (95% CI 1.30–1.40) for females and RR 1.31 (95% CI 1.21–1.43) for males, respectively (Fig 2A). For the relative risk of developing coronary artery diseases (CAD) the results appear similar to those of stroke (Fig 2B). Females with RA had a RR of 1.65 (95% CI 1.54–1.76) while men with RA had a RR of 1.55 (95% CI 1.41–1.69), which again is not significantly different (p = 0.27). Heterogeneity was low (I^2^ < 25%) to moderate (I^2^ < 50%) in all analyses above. When CVD was used as combined outcome measure, women and men with RA also have a similar increased risk: RR 1.56 (95% CI 1.49–1.62) in women and RR 1.50 (95% CI 1.41–1.60) in men, respectively (Fig 2C). Finally, mortality due to CVD was increased in RA patients, in women: RR 1.51 (95% CI 1.19–1.92) and in men: RR 1.44 (95% CI 0.97–2.14), (p = 0.84 for the gender comparison), and overall: RR 1.57 (95% CI 1.39–1.79), p < 0.00001. However, heterogeneity among these three (males) and four (females) studies was high (I^2^ > 95%) in total and after stratification for gender (no forest plots shown).

### Influence of age on CV events

**Fig 3:**
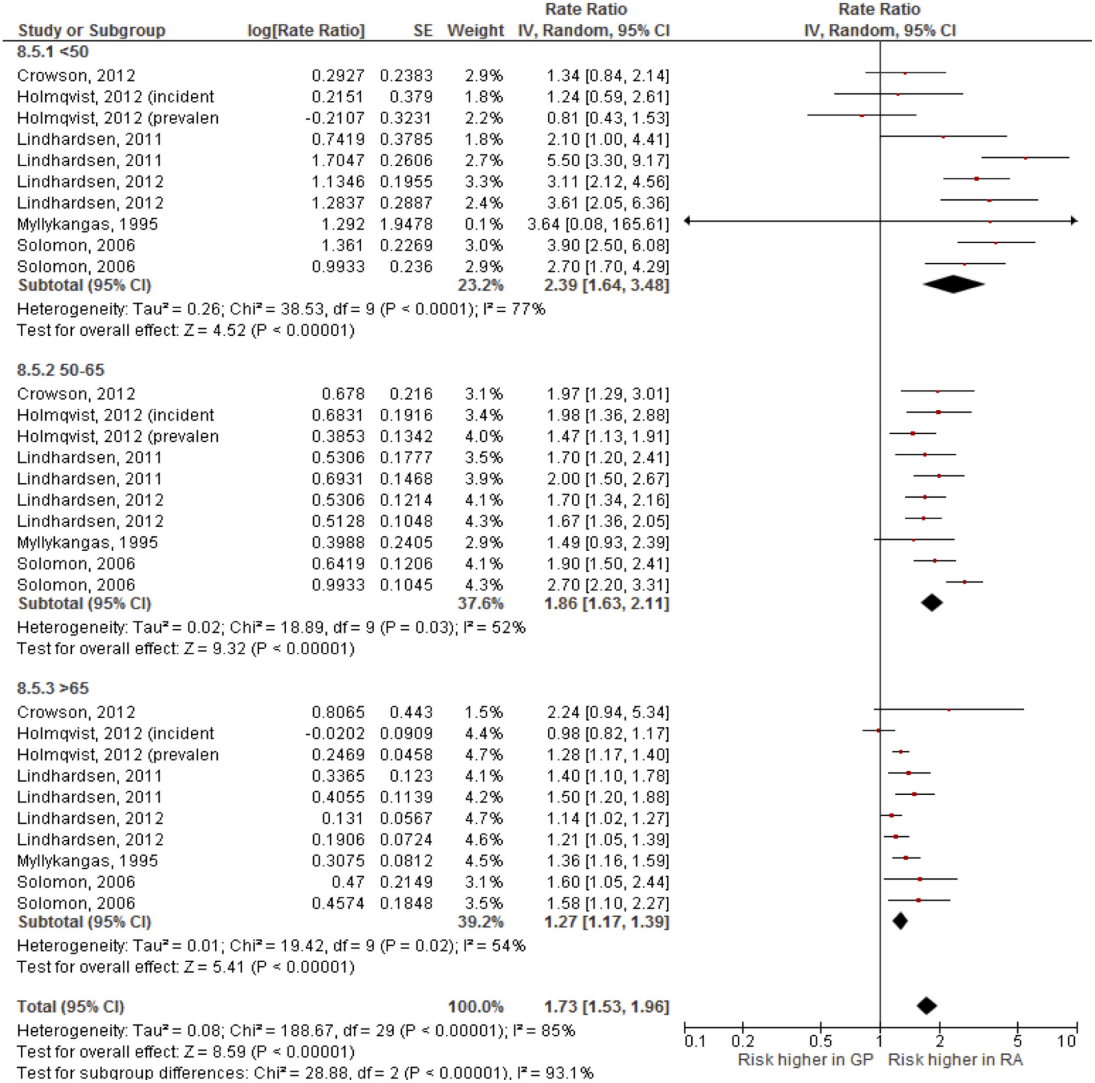
The effect of age on a”pooled” CVD outcome in patients with RA. The groups are stratified based on the age in three subgroups: <50 years, 50–65 years and >65 years, respectively.

There were five studies in which CVD relative risks were stratified by age group (S1 Table), reporting on CVD (1 study), stroke (2 studies), CAD (1 study) and mortality in females (1 study) and we pooled the results of these six studies to analyze the influence of age on the relative risk for CVD in patients with RA as compared to people from the general population (Fig 3). In the previous analyses there appeared to be no statistically or clinically relevant differences in relative risk between RA males and RA females. Therefore, and as only 3 studies had stratified their results by age-as-well-as-gender, the data of males and females were pooled. The included studies usually used different age limits to define age groups in comparing RA patients with people from the general population. Given the data, it was possible to pool according to three age categories: younger than 50 years, between 50 and 65 years, and older than 65 years, respectively.

As shown in Fig 3, the relative risk for CVD was highest in the youngest RA patients: RR 2.59 (95% CI 1.77–3.79), whereas older RA patients had the lowest relative risk when compared to the general population: RR 1.27 (95% CI 1.16–1.38). There appeared to be a significant age effect (p < 0.00001) while at the same time heterogeneity was considerable (I^2^>50%) within age groups and for the overall effect.

## Discussion

According to the results of this meta-analysis, the well-known increased risk for cardiovascular disease of RA patients, as compared to the general population, is dependent on age, but does not depend on gender. That is to say, the relative risk on CV disease appears to be equal for males and females, the relative risk was just only slightly, non-significantly, higher in females. It also appeared that the relative risk on CVD in RA was age dependent, with relatively young RA patients (<50 years) bearing the highest relative risk and the elder RA patients (>65 years) having the lowest relative risk. Consequently, it appears that RA increases the risk on CVD in all patients, but the relative risk is largest in younger patients, for males and females alike. Due to lack of information and due to heterogeneity between studies, we were not able to pool absolute risks in this meta-analysis. Notably, relative risks are better generalizable than absolute risks and risk differences. For that same reason, it was refrained from calculating a number-needed-to-screen, a number-needed-to-treat analogue, from the inverse of the risk differences [27]. From the quality appraisal it appeared that the quality of the studies is quite high. A main finding was that for two studies (references 23 and 24), it was not clear whether or not residual confounding beyond age and gender was present, which was reflected in the quality appraisal score. Because of this uncertainty and high overall quality it may be regarded that the risk of bias for the results of the meta-analysis is quite low.

Having increased CV risk has bearings on CV prevention in RA. However, CV risk calculators used in the general population are of a limited utility in RA. They mostly underestimate the CV risk, especially in the group of patients originally classified as having a low or intermediate risk [3]. The results of the present study are the first that can offer a simple explanation for this discrepancy: risk of CVD in the RA population is distributed differently with regard to age, as compared to the general population. Therefore, it is likely that the true risk on CVD is misjudged especially in younger RA patients. The European League Against Rheumatism (EULAR) has previously developed specific recommendations in order to better estimate the CV risk in RA. Accordingly, a multiplying factor of 1.5 is applied to each RA patient having at least two of the following features: a disease duration longer 10 years, extra-articular manifestations or RF/aCCP positivity [28]. Yet, the performance of this adapted risk calculator is still limited for RA patients [3]. Moreover, other factors may limit the utility of such a score: extra-articular features are more and more sporadic given the new treatment strategies and possibilities, while disease duration and serology is not firmly associated with higher risk [29, 30]. We recently attempted to recalibrate the SCORE risk algorithm by re-weighing the included traditional CVD risk factors and to adapt it by adding other potential predictors to the existing SCORE variables [4]. Even this adaptation did not provide sufficient improvement in the calculation of the risk for future CVD events in these patients.

Therefore, instead of searching for new cardiovascular risk factors specific for RA (inflammatory markers, etc), improving the evaluation of CV risk in this group of patients might have perhaps a simpler solution. Based on the results of the present analysis we suggest that age must gain a different weight and meaning in RA with respect to the future risk of developing CV events. There may be at least two strategies to translate this into practice. The first one implies the change of age’s weight in the current CV risk calculators, in order to redistribute patients from lower to higher risk categories. This would result into a larger number of RA patients entitled to receive primary prevention measures/therapies. This strategy may however lead to over-diagnosis, over-treatment and might raise the costs unnecessary. Yet, no data is available to confirm or infirm this strategy. We currently undergo a study to look for the cost-effectiveness of screening for CV risk factors using various risk calculators in a primary prevention setting. One such calculator is recommended by the Dutch National Guidelines for CV risk management, and suggest adding 15 years to the age of all RA patients [31]. If our study will provide evidence for a cost-effective strategy despite adding 15 years to the age of patients, this will support our suggestion of modifying age’s weight in the current algorithms. The second approach through which the results from the present study might improve the identification of RA patients with high CV risk, would take into account the natural course of atherosclerosis, as main determinant of CVD in RA. Accordingly, in RA patients due to the chronic inflammation atherosclerosis is likely to develop earlier at younger ages as compared to general population and further evolves at accelerated levels [32, 33]. Therefore, there is very likely a window of opportunity in younger RAs to treat atherosclerosis in order to prevent CVD at older ages. This is also supported by recent observations of altered lipid profiles before disease onset [34]. Consequently, the second strategy may be the use of an extra measurement (besides the current risk calculator), e.g. carotid artery ultrasound, coronary artery calcification index etc, in order to identify atherosclerosis in younger RA patients with low CV risk according to current risk algorithms. Previous studies have shown that such an approach might be followed by a relevant redistribution of patients from lower to higher risk categories [35]. Therefore, as alternative strategy, we suggest that young RA patients ending up in the low CV risk group should receive further investigations on the presence/absence of atherosclerosis.

Interpreting the evidence from observational studies requires caution. Some of the included studies had a retrospective design, which may contribute to bias. However, all included studies were of reasonable to good quality, according to the quality ratings using the Newcastle-Ottawa Quality Assessment Scale. To their strength, most of the studies used national or regional registries for diseases and all retrieved the RA sample and the general population sample from the same source population. Most studies used incidence rate ratios to compare occurrence of CVD in RA and general populations, but some used standardized mortality rations or standardized morbidity ratios. Using the inverse variance method for meta-analysis may compensate for these differences. For most of the included studies, the age and gender association with CVD was not the primary objective, which was why not all of them showed results stratified by age and gender. However, all existing studies providing these data have been included in the analysis, and therefore our results are likely to be the most representative on this topic. The most important limitation we regard, is the fact that not all the studies investigated the same CV end-points, while some studies used a combined CV outcome, which led to a relative low number of studies included in analysis per type of event (stroke, CAD, etc).

To corroborate the results of this meta-analysis, the analyses could be repeated, stratified by age and gender, for specified outcomes, in several large databases, after which the results may be combined in meta-analysis. Maybe even individual patient data meta-analysis can be used.

## Conclusions

Male and female patients with RA have a similar relative risk on CVD. Interestingly, in the youngest RA patients the relative risk on CVD is highest, independently of gender. These results may help to raise clinical attention to the risk groups, especially the younger RA patients, to improve the accuracy of predicting CV risk in RA and eventually diminish the incidence of and death due to CVD in this population.

## Supporting Information

S1 Table Cardiovascular disease outcomes by sex and age in Rheumatoid Arthritis cohort studies. (DOCX)

## Abbreviations

aCCP: anti-cyclic cytrullinated peptide antibodies
CAD: coronary artery disease
CV: cardiovascular
CVD: cardiovascular diseases
EULAR: the European league against rheumatism
RA: rheumatoid arthritis
RF: rheumatoid factor
