# Supplementary material for: Rheumatoid Arthritis Disadvantages Younger Patients for Cardiovascular Diseases: A Meta-Analysis
Source: PLoS One. 2016 Jun 16;11(6):e0157360. doi: 10.1371/journal.pone.0157360 (PMC4911000; doi:10.1371/journal.pone.0157360)
Supplement: S1 Table — (DOCX) [file pone.0157360.s001.docx]

Supplementary Table 1. Cardiovascular disease outcomes by sex and age in Rheumatoid Arthritis cohort studies.

| **Reference number** | **Author, year** | **Sex** | **Age** | **SMR** | **IRR** | **Mortality** | **Morbidity and mortality** | | | |
| --- | --- | --- | --- | --- | --- | --- | --- | --- | --- | --- |
|  |  |  |  |  |  | **CVD** | **CAD/stroke** | **CAD** | **Stroke** | **CVD** |
| 20 | Holmqvist, 2013 | F |  |  | • |  |  |  | 1.28 (1.14-1.43) |  |
|  |  | M |  |  | • |  |  |  | 1.31 (1.13-1.52) |  |
|  |  |  | 16-52 |  | • |  |  |  | 0.81 (0.43-1.54) |  |
|  |  |  | 53-62 |  | • |  |  |  | 1.47 (1.13-1.90) |  |
|  |  |  | 63-71 |  | • |  |  |  | 1.36 (1.14-1.62) |  |
|  |  |  | 72-94 |  | • |  |  |  | 1.25 (1.12-1.41) |  |
|  |  | F |  |  | • |  |  |  | 1.16 (0.95-1.42) |  |
|  |  | M |  |  | • |  |  |  | 1.04 (0.82-1.32) |  |
|  |  |  | 16-52 |  | • |  |  |  | 1.24 (0.59-2.58) |  |
|  |  |  | 53-62 |  | • |  |  |  | 1.98 (1.36-2.89) |  |
|  |  |  | 63-71 |  | • |  |  |  | 1.04 (0.77-1.40) |  |
|  |  |  | 72-94 |  | • |  |  |  | 0.95 (0.76-1.18) |  |
| 16 | Lindhardsen, 2012 | F |  |  | • |  |  |  | 1.33 (1.22-1.46) |  |
|  |  | M |  |  | • |  |  |  | 1.34 (1.19-1.51) |  |
|  |  | F | <50 |  | • |  |  |  | 3.11 (2.12-4.57) |  |
|  |  |  | 50-65 |  | • |  |  |  | 1.67 (1.36-2.06) |  |
|  |  |  | >65 |  | • |  |  |  | 1.14 (1.02-1.26) |  |
|  |  | M | <50 |  | • |  |  |  | 3.61 (2.05-6.36) |  |
|  |  |  | 50-65 |  | • |  |  |  | 1.70 (1.34-2.15) |  |
|  |  |  | >65 |  | • |  |  |  | 1.21 (1.05-1.40) |  |
| 17 | Lindhardsen, 2011 | F |  |  | • |  |  | 1.7 (1.4-2.0) |  |  |
|  |  | M |  |  | • |  |  | 1.6 (1.4-1.9) |  |  |
|  |  | F | <50 |  | • |  |  | 5.5 (3.3-9.2) |  |  |
|  |  |  | 50-65 |  | • |  |  | 1.7 (1.2-2.3) |  |  |
|  |  |  | >65 |  | • |  |  | 1.4 (1.1-1.7) |  |  |
|  |  | M | <50 |  | • |  |  | 2.1 (1.0-4.2) |  |  |
|  |  |  | 50-65 |  | • |  |  | 2.0 (1.5-2.6) |  |  |
|  |  |  | >65 |  | • |  |  | 1.5 (1.2-1.9) |  |  |
| 14 | Semb, 2010 | F |  |  | • |  |  | 1.90 | 1.60 |  |
|  |  | M |  |  | • |  |  | 1.61 | 1.61 |  |
| 21 | Bergström, 2009 | F |  | • |  |  |  | 2.19 (1.25-3.55) | 0.75 (0.20-1.93) | 1.65 (1.05-2.48) |
|  |  | M |  | • |  |  |  | 2.40 (1.04-4.73) | 1.15 (0.14-4.14) | 1.41 (0.61-2.77) |
|  |  | F |  | • |  |  |  | 2.24 (1.30-3.59) | 1.53 (0.76-2.74) | 1.86 (1.24-2.69) |
|  |  | M |  | • |  |  |  | 1.81 (0.66-3.93) | 1.45 (0.39-3.71) | 1.40 (0.64-2.66) |
| 18 | Solomon, 2006 | F |  |  | • |  | 1.6 (1.5-1.7) |  |  |  |
|  |  | M |  |  | • |  | 1.6 (1.5-1.8) |  |  |  |
|  |  | F | 18-49 |  | • |  | 3.9 (2.5-6.0) |  |  |  |
|  |  |  | 50-64 |  | • |  | 2.7 (2.2-3.2) |  |  |  |
|  |  |  | 65-74 |  | • |  | 2.0 (1.7-2.2) |  |  |  |
|  |  |  | >74 |  | • |  | 1.3 (1.2-1.4) |  |  |  |
|  |  | M | 18-49 |  | • |  | 2.7 (1.7-4.3) |  |  |  |
|  |  |  | 50-64 |  | • |  | 1.9 (1.5-2.3) |  |  |  |
|  |  |  | 65-74 |  | • |  | 1.9 (1.7-2.2) |  |  |  |
|  |  |  | >74 |  | • |  | 1.3 (1.1-1.5) |  |  |  |
| 15 | Turesson, 2004 | F |  | • |  |  | 1.55 (1.06-2.19) | 1.80 (1.03-2.56) | 1.12 (0.58-1.95) |  |
|  |  | M |  | • |  |  | 1.69 (1.06-2.57) | 1.72 (0.96-2.83) | 1.37 (0.59-2.70) |  |
| 22 | Goodson, 2002 | F |  | • |  | 1.10 (0.67-1.69) |  |  |  |  |
|  |  | M |  | • |  | 0.70 (0.36-1.22) |  |  |  |  |
| 23 | Wallberg, 1997 | F |  | • |  |  |  | 1.68 | 1.39 | 1.54 |
|  |  | M |  | • |  |  |  | 1.41 | 0.57 | 1.36 |
| 24 | Myllykangas, 1995 | F |  | • |  | 1.34 |  |  |  |  |
|  |  | F | 15-49 | • |  | 3.64 |  |  |  |  |
|  |  |  | 50-59 | • |  | 1.29 |  |  |  |  |
|  |  |  | 60-64 | • |  | 1.55 |  |  |  |  |
|  |  |  | 65-69 | • |  | 1.45 |  |  |  |  |
|  |  |  | 70-74 | • |  | 1.68 |  |  |  |  |
|  |  |  | 75-79 | • |  | 1.40 |  |  |  |  |
|  |  |  | 80-84 | • |  | 1.24 |  |  |  |  |
|  |  |  | >84 | • |  | 1.09 |  |  |  |  |
| 19 | Norton, 2013 | F |  |  | • |  |  | 1.42 (1.15-1.76) | 1.34 (1.02-1.77) |  |
|  |  | M |  |  | • |  |  | 1.83 (1.48-2.27) | 0.94 (0.62-1.42) |  |
| 25 | Watson, 2003 | F |  |  | • | 1.5 (1.4-1.6) |  | 1.6 (1.5-1.8) | 1.4 (1.3-1.5) | 1.5 (1.4-1.6) |
|  |  | M |  |  | • | 1.4 (1.2-1.6) |  | 1.4 (1.3-1.6) | 1.4 (1.2-1.6) | 1.4 (1.3-1.5) |
| 26 | Thomas, 2003 | F |  | • |  | 1.97 (1.93-2.01) |  |  |  |  |
|  |  | M |  | • |  | 2.07 (2.01-2.13) |  |  |  |  |
